# Timothy Grass Pollen-Specific Immunoglobulin E in Nasal Secretions During Natural Allergen Exposure and After a Nasal Provocation Test in Patients with Suspected Local Allergic Rhinitis

**DOI:** 10.3390/arm94040056

**Published:** 2026-08-03

**Authors:** Mohamad Mahdi Mortada, Waleed Aman Ur Rahman, Alaa Sherri, Gabriela Pawlak, Krystian Kowalski, Anna Piłat, Edyta Pietrowska, Marta Popławska, Iwona Dziembała-Gładysz, Barbara Majkowska-Wojciechowska, Marcin Kurowski

**Affiliations:** 1 Department of Immunology and Allergy, Medical University of Łódź, 92-213 Łódź, Poland; mohamadmahdi.mortada1@student.umed.lodz.pl (M.M.M.); waleed.amanurrahman@student.umed.lodz.pl (W.A.U.R.); iwona.dziembala-gladysz@umed.lodz.pl (I.D.-G.);; 2 Department of Rheumatology, Medical University of Łódź, 90-419 Łódź, Poland; 3 Department of Immune Metabolism, Swiss Institute of Asthma and Allergy Research (SIAF), CH-7265 Davos, Switzerland; 4 Medical Immunological Diagnostics Laboratory, Department of Laboratory Diagnostics, Central Teaching Hospital of the Medical University of Łódź, 92-213 Łódź, Poland; gabriela.pawlak@csk.umed.pl (G.P.); krkowalski@csk.umed.pl (K.K.); a.pilat@csk.umed.pl (A.P.); 5 Biobank HARC, Medical University of Łódź, 92-213 Łódź, Poland

**Keywords:** specific Immunoglobulin E (IgE), nasal provocation test, grass pollen allergen, local allergic rhinitis, and nasal mucosa

## Abstract

**Highlights:**

**What are the main findings?**

- Nasal provocation test was positive in 46% of suspected patients with local allergic rhinitis, among whom significantly higher total nasal symptoms and Visual analog scores were noted post-provocation.
- Our study showed a higher relative sIgE concentration (calculated as sIgE/total IgE) in nasal secretions after provocation in patients with confirmed LAR diagnosis.

**What are the implications of the main findings?**

- The positive nasal provocation (NPT) results in almost 50% of the suspected LAR patients, suggesting that NPT is, to date, the gold standard diagnostic tool in LAR diagnosis, in whom an allergic response may not be easily detected through conventional tests.
- The notably elevated grass pollen sIgE to total IgE ratio seen after provocation in confirmed LAR patients suggests that the assessment of relative sIgE level can help in enhancing the accuracy of LAR diagnosis and offers additional proof of allergic inflammation at the local level, especially in patients with inconclusive systemic allergy tests such as skin prick testing and serum IgE level.

**Abstract:**

Introduction: Allergic rhinitis (AR) imposes a substantial burden on individuals worldwide. Local allergic rhinitis (LAR) patients exhibit symptoms similar to those of AR without a positive skin prick test (SPT) or the presence of sIgE specific to one or more allergens. Our study evaluated specific and total grass pollen IgE in the nasal secretions of patients with suspected LAR, examining the effects of natural pollen exposure and nasal provocation testing (NPT). Methods: Twenty-nine subjects (18 with suspected LAR and 11 with AR) were included in this study. The total nasal symptom score (TNSS) and visual analog scale (VAS) were used for subjective assessment. NPT was performed using grass pollen allergen. Nasal lavage was used to obtain nasal secretions, and the levels of sIgE and total IgE were measured. Results: During the exposure vs. the off-exposure period, the TNSS and VAS were significantly higher in LAR (*p* < 0.0001 and *p* = 0.0061, respectively). sIgE and total IgE were significantly higher in the AR group than in the LAR group during the exposure period (*p* = 0.0008 and *p* = 0.0491, respectively). LAR diagnosis was confirmed in seven subjects. TNSS and VAS scores were higher after a positive NPT. The median level of sIgE was lower in the negative and positive NPT. A higher median total IgE level was observed post-provocation in both groups. A significantly higher median relative sIgE level was noted after positive and negative provocation (*p* = 0.0156 and *p* = 0.0143, respectively). Conclusions: The assessment of local sIgE levels in nasal secretions can be used as a method for the diagnostic workup of patients with suspected LAR. Achieving a well-defined and standardized protocol for the assessment of sIgE concentrations in nasal secretions of LAR is a goal of future larger studies.

## 1. Introduction

Allergic rhinitis (AR) brings a substantial burden to individuals worldwide, with prevalence reaching up to 20% in the adult population in Europe [1]. The clinical manifestations of AR, including rhinorrhea, sneezing, nasal itching, and congestion, negatively impact the daily lives of affected individuals. AR is suspected based on the previously mentioned symptoms and confirmed by a positive skin-prick test and/or serum-specific immunoglobulin E (sIgE) assessment [2]. Thus, an IgE-mediated reaction to a variety of inhaled allergens is documented and clinically confirmed. The term „local allergic rhinitis” (LAR) has been coined with the intention to describe situations where patients show symptoms similar those of AR without, however, the presence of sIgE confirmed either through a positive skin prick test (SPT) or the presence of sIgE to one or more allergens [3,4]. Such a situation poses a considerable diagnostic and therapeutic challenge. Clinical symptoms currently classified as LAR have long been considered manifestations of non-allergic rhinitis (NAR). Additionally, the coexistence of both AR and LAR has been observed in a subset of affected subjects and is referred to as dual allergic rhinitis (DAR) [5]. Although epidemiological studies on LAR are limited, prevalence estimates range from 20% to 60% and 3% to 68% in adult and pediatric populations, respectively [3,6,7]. However, these ranges differ substantially across studies, reflecting differences in demographics and methodologies. The main allergens studied in this context are house dust mites and grass pollen allergens, and, to a lesser extent, molds and animal allergens [3]. The pathophysiological mechanisms underlying LAR are not yet fully elucidated. The primary mechanism is a Th2 reaction, including IgE-mediated reactions and the activation and activity of several mediators and proteins, mainly tryptase, eosinophil cationic protein (ECP), thymic stromal lymphopoietin (TSLP), interleukin (IL-) 5, IL-10, IL-13, IL-17, monocyte chemoattractant protein (MCP)-4, eotaxin-3 (CCL26), and transforming growth factor (TGF-) [3,6,7,8]. As a significant number of LAR, as well as DAR patients, are still either underdiagnosed or misdiagnosed, this usually delays the start of the treatment, thus putting those subjects at a greater risk of possible conversion of the local reaction into a systemic disease with an increase in rhinitis symptom severity over the years of disease progression [4]. NPT is the “gold standard” diagnostic test for confirming LAR [2]. Nevertheless, it is not only time-consuming but also requires trained personnel and full patient cooperation to be performed correctly. The assessment of sIgE in nasal secretions of suspected LAR subjects is an additional diagnostic tool [9]. Allergen immunotherapy (AIT) shows promising results in treating LAR and DAR subjects; however, this management option is not yet included in the treatment guidelines for affected individuals [10,11]. Our study aimed to assess the clinical symptoms and local mucosal sIgE presence post-provocation and during the exposure season to grass pollen allergen (g6 Allergen) in subjects with suspected LAR and AR.

## 2. Materials and Methods

### 2.1. Subjects’ Recruitment

Subjects were recruited between June 2022 and July 2024. All recruited subjects received detailed explanations of the steps to be performed during NPT and sample collection and provided written consent to participate in the study. The study was approved by the Bioethics Committee of the Medical University of Lodz (decision no. RNN/76/22/KE dated 10 May 2022).

Participants were recruited from outpatients followed up at the tertiary academic allergy clinic in central Poland, in the metropolitan area of approximately 700,000 inhabitants. They were permanent residents of this area throughout the study period. Patients with suspected allergic rhinitis underwent a diagnostic assessment that included a detailed medical history focused on rhinitis symptoms, as well as IgE determination using SPT and serum sIgE measurement. LAR was suspected based on the presence of symptoms mainly following grass pollen exposure, a negative SPT result, and the absence of serum sIgE to timothy grass pollen allergens and to other potentially clinically relevant seasonal allergens whose presence overlaps with that of grass pollen allergens in the study area (oak tree, mugwort, *Alternaria*, *Cladosporium*). AR subjects were selected based on the presence of seasonal symptoms, positive SPT, and positive serum sIgE assessment.

The following exclusion criteria were applied.

- Lack of informed consent for participation
- Concomitant nasal polyposis, chronic rhinosinusitis, nasal septum deviation, or any other upper airway condition that could affect the outcomes of the study as judged by the investigators.
- Uncontrolled asthma, in line with current national and international guidelines [12]
- Clinically relevant, confirmed IgE-mediated sensitization to seasonal and/or perennial aeroallergens other than grass pollen allergens
- Respiratory infection within 4 weeks before the day of NPT and nasal lavage collection
- Inability or unwillingness to withdraw medication that could interfere with the performance of the study procedures or influence the results
- Any other conditions that, in the investigators’ judgment, could influence the outcome of the procedures or interfere with patients’ ability to participate
- Pregnancy or breastfeeding

Participants were asked to refrain from using the following medications during the indicated period before the NPT and nasal lavage sampling procedure.

- Systemic and nasal glucocorticosteroids—4 weeks
- Systemic antihistamines—2 weeks
- Nasal decongestants—2 weeks
- Intranasal antihistamines—3 days

### 2.2. Skin Prick Tests (SPT) and Serum IgE Assessment

Skin prick tests were performed using a panel of airborne allergens: *D. pteronyssinus, D. farinae,* alder, hazel, birch, grass pollen mix, rye, mugwort, cat, dog, *Alternaria*, and *Cladosporium* (Diater, Leganés, Spain). The wheal and flare sizes were measured after 15 min, and wheals with a diameter ≥3 mm were considered positive. Histamine hydrochloride (1 mg/mL) and buffer solution were used as positive and negative controls, respectively. Serum sIgE levels to airborne allergens were assessed using EUROLINE allergy diagnostic profiles (EUROIMMUN Medizinische Labordiagnostika AG, Lübeck, Germany). Additionally, sIgE levels in grass pollen allergen extracts were measured using the ImmunoCAP immunoassay (Thermo Fisher Scientific, Uppsala, Sweden). Serum sIgE concentrations exceeding the threshold of 0.35 kUA/L were considered positive, as indicated by the manufacturers of the diagnostic tools.

### 2.3. Total Nasal Symptom Score (TNSS) and Visual Analog Scale (VAS)

TNSS is the sum of the individually measured scores of (1) nasal congestion, (2) sneezing, (3) nasal itching, and (4) rhinorrhea using a four-point scale (0–3), where 0 indicates no symptoms, 1 for mild symptoms, 2 for symptoms that are bothersome but tolerated by the subject, and 3 for severe symptoms. TNSS is calculated by summing the scores for each symptom, with a maximum of 12. VAS is a 100 mm scale that ranges from “no symptoms” to “worst symptoms ever” applied to the overall intensity of the symptoms associated with rhinitis.

### 2.4. Nasal Provocation Test (NPT)

NPT was performed outside the exposure period to grass pollen allergen using dissolved immunotherapy tablets (Grazax, ALK, Copenhagen, Denmark). In accordance with the protocol published by Eifan et al. [13]. In brief, two tablets of Grazax (75,000 SQ-T of purified *Phleum pratense* lyophilisate) were dissolved in 1.5 mL of saline solution, which gives a stock solution at a concentration of 100,000 SQ-T/mL, then 0.5 mL of the stock solution was added to 1 mL of saline in a second sterile container, which gives a solution of 33,333 SQ-T/mL. A further 0.5 mL of this solution was added to 0.5 mL of saline in a third container, yielding a concentration of 16,667 SQ-T/mL of *Phleum pratense* extract. Subsequently, two 100 μL sprays were administered to the patient using a nasal applicator.

The following procedures were performed 15 min after provocation:

(1) Self-completion of the TNSS/VAS questionnaires by the participants (also completed before NPT)
(2) Physician’s assessment of the participants through anterior rhinoscopy regarding any of the following symptoms: swelling, redness, or nasal discharge.
(3) Acquisition of the nasal secretions through the nasal lavage (see Section 2.5)

A positive NPT was noted by either the presence of nasal symptoms subjectively assessed by the subjects through completing the TNSS/VAS questionnaire according to the European Academy of Allergy and Clinical Immunology (EAACI) position paper on the standardization of nasal allergen challenges [14] and/or through the presence of symptoms, such as swelling, redness, and discharge, as assessed by the physician using anterior rhinoscopy. In our study, we did not use objective assessment tools such as peak nasal inspiratory flow (PNIF), acoustic rhinometry (AcRh), active anterior rhinomanometry (AAR), and 4-phase rhinomanometry (4 PR), which are also recommended by the abovementioned position paper. This approach was chosen because the study was designed and planned during the last phase of the COVID-19 pandemic, and using a PNIF measurement available at the study site might have raised concerns about the risk of infection transmission. To maintain uniformity in the study procedures, PNIF was not added in subsequent years, and the NPT outcome was assessed solely based on the patient’s subjective assessment, as one of the possible criteria listed in the EAACI position paper.

### 2.5. Nasal Secretions Acquisition

Nasal secretions were collected at the following time points:

- In the LAR group, during the grass pollen exposure period, and before and after NPT in the off-exposure period
- In the AR group, during the grass pollen exposure period

Nasal sampling was performed using the noninvasive technique of nasal lavage, as previously described [15]. In the nasal lavage technique, the participant was asked to sit on a chair and lean forward. A volume of 5 mL of saline solution was administered to one nostril via a syringe connected to an olive. After 10 min, the solution was retrieved, and the collected volume was recorded. Nasal secretions were acquired before and after NPT, as well as during the season of increased exposure to grass pollen allergen.

In Poland, the grass pollination season usually starts in mid-May. It may last for more than 100 days, while for 52 days, pollen counts exceed the threshold value of 20 grains/m^3^ considered as the one inducing symptoms in most sensitized allergic individuals [16]. Subsequently, the samples were centrifuged at 3000 rpm at 4 °C for 15 min and stored at −70 °C until further analysis. The dilution of nasal secretion by lavage fluid was estimated according to a published formula [17] as follows: dilution factor (DF) = [urea serum] × 1.2/ [urea in NLF]. The sIgE concentration in the collected nasal secretions, as well as the total IgE concentration, was calculated by multiplying the DF by the NLF concentration. Urea levels in serum and nasal secretions were measured spectrophotometrically using a commercially available kit (Cell Biolabs, Inc., San Diego, CA, USA).

### 2.6. Local Specific Immunoglobulin E (sIgE) and Total IgE

Concentrations of total and timothy grass pollen allergen-sIgE in nasal secretions were quantitatively measured using an ImmunoCAP immunoassay (Thermo Fisher Scientific, Uppsala, Sweden). Cut-off value for sIgE was 0.10 kUA/L, and for total IgE − 2.0 kU/L, as per the manufacturer’s data.

### 2.7. Grass Pollen Count Measurement

Aerobiological data on airborne pollen counts of grasses of the *Poaceae* family in the Łódź area during the grass pollination seasons in the years 2022 through 2024 were obtained using a volumetric pollen trap of the Hirst design, as previously described [18,19,20], located in the downtown area of the city (ca. 700,000 inhabitants) where the recruited patients resided. Briefly, air was sucked into the trap at a rate of 10 L/min through an orifice of dimensions 2 mm × 14 mm. The air behind the orifice flowed over the rotating drum, moving at 2 mm/h and coated with an adhesive transparent tape. The tape used for capturing pollen grains was replaced weekly. Pollen grains were counted using a light microscope at 400× magnification. Following the removal of the tape from the trap, it was cut into segments corresponding to 24 h periods. Pollen grains were counted, and the results were expressed as daily mean pollen concentration per cubic meter of air according to the formula recommended by the International Association for Aerobiology. The *Poaceae* genera whose airborne pollen are predominant in Poland include: *Phleum pratense* L., *Dactylis glomerata* L., *Festuca elatior* L., *Lolium perenne* L., *Poa pratensis* L, and *Anthoxanthum odoratum* L. [21,22].

### 2.8. Statistical Analysis

Statistical analysis was performed using GraphPad Prism version 10 for macOS (GraphPad Software, Boston, MA, USA). The normality of the data distribution was tested using the Shapiro–Wilk test. The nonparametric Mann–Whitney U test or Wilcoxon matched-pairs signed-rank test was used to assess the significance of differences between the groups. Differences were considered statistically significant at *p* < 0.05. The rank-biserial correlation coefficient (*r_rb_*) was employed to quantify the non-parametric effect size of the group differences, using an online tool (available from: https://www.statscalculators.com/, accessed 19 July 2026). Effect sizes were classified as small (*r_rb_* ≥ 0.10), moderate (*r_rb_* ≥ 0.30), or large (*r_rb_* ≥ 0.50) following standard thresholds.

## 3. Results

### 3.1. Descriptive Data

A total of 29 subjects were included in our study (Table 1). A total of 18 suspected LAR subjects were included, 11 females and seven males. The median age of female participants in the LAR group was 47 years (range, 19–69), while for male participants, it was 32 years (range, 23–57). A total of 11 AR participants were included, five females and six males. The median age of female participants in the AR group was 20 years (range, 18–68), while for male participants, the median age was 27 years (range, 19–44).

### 3.2. Specific and Total IgE in Nasal Secretions During the Grass Pollen Exposure Period and Off-Exposure Period

**Figure 1:**
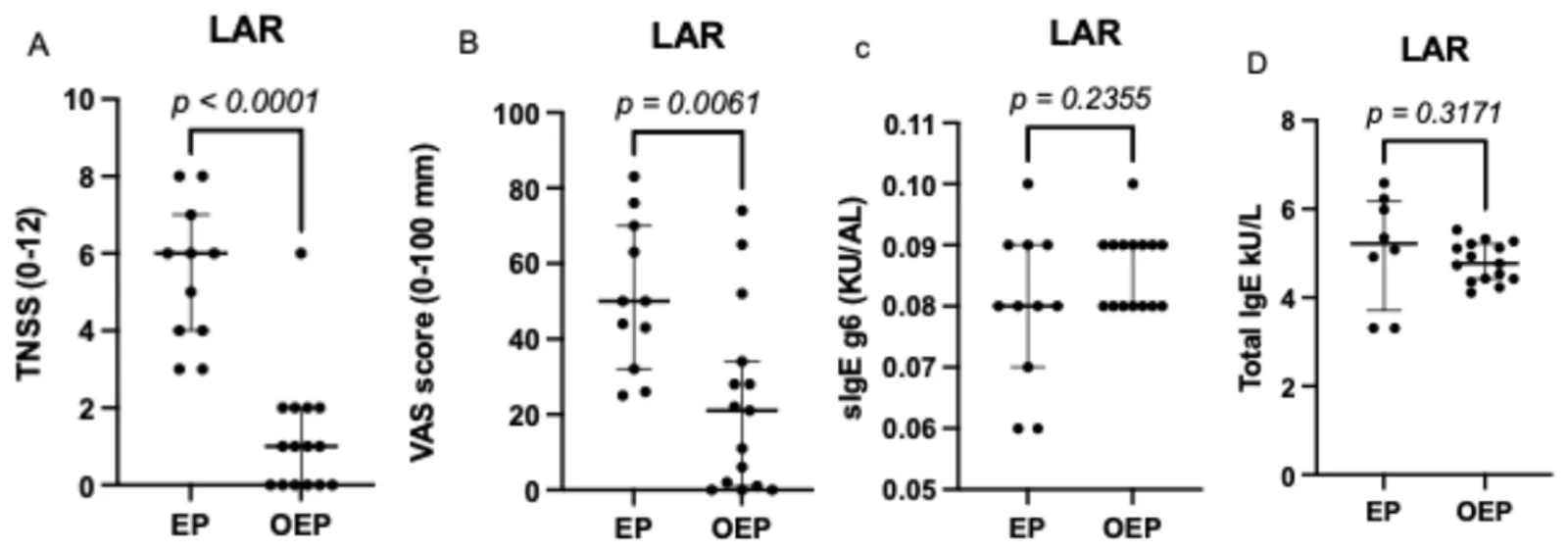
Comparison of (**A**) TNSS, (**B**) VAS, (**C**) sIgE g6, and (**D**) total IgE among LAR subjects during the exposure period vs. outside the exposure period. LAR, Local allergic rhinitis. TNSS, Total nasal symptom score; VAS, Visual analog scale; sIgE, specific immunoglobulin E; g6, Timothy grass. EP, Exposure period. OEP, Outside exposure period. Results are presented as median + interquartile range (IQR). One dot represents one assessment.

**Figure 2:**
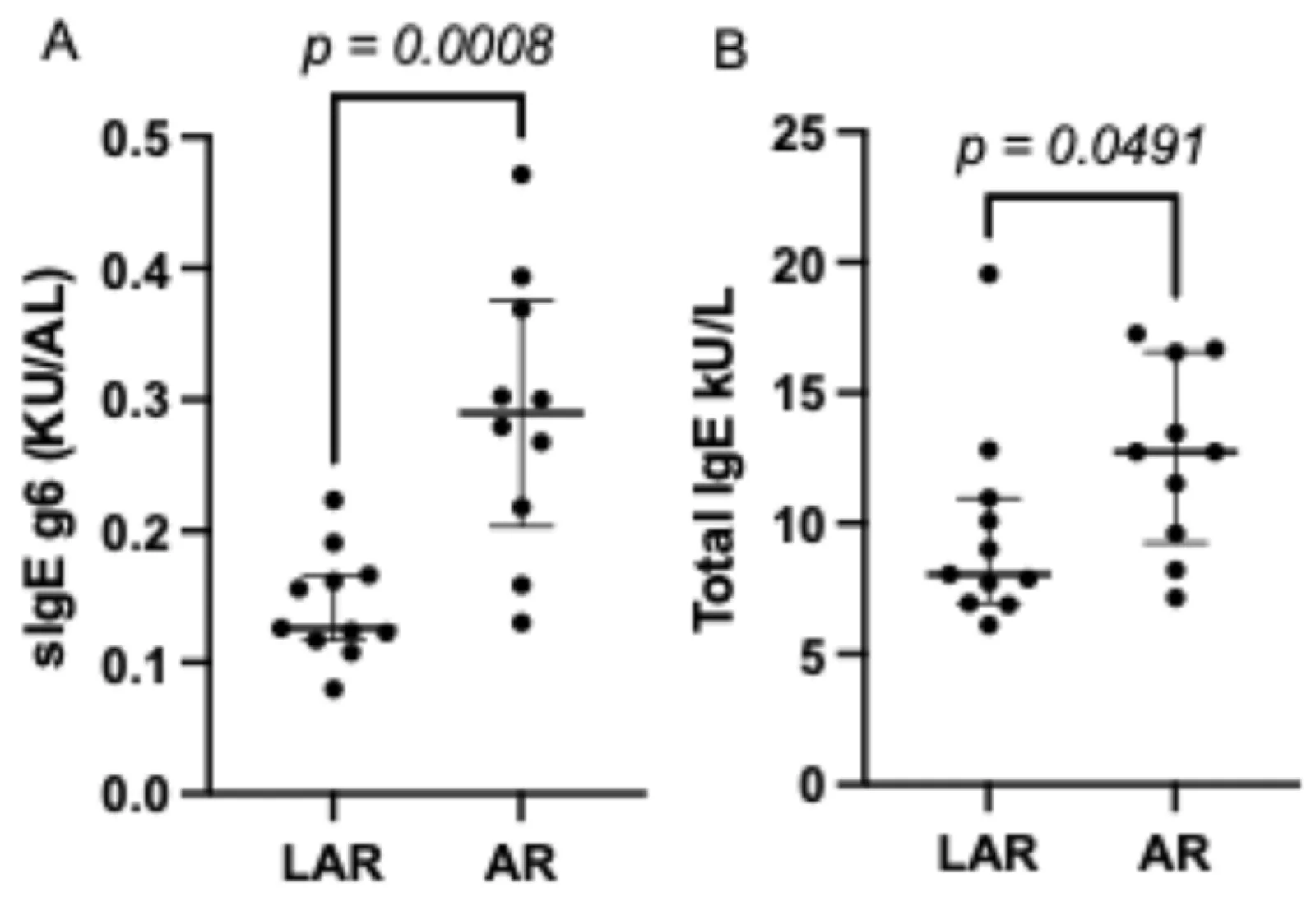
Comparison of (**A**) sIgE to timothy grass pollen allergen extract (g6) and (**B**) total IgE between LAR and AR subjects during the allergen exposure period. sIgE, specific immunoglobulin E; g6, Timothy Grass; AR, Allergic Rhinitis; LAR, Local allergic rhinitis. Results are presented as median + interquartile range (IQR). One dot represents one subject.

In the suspected LAR group, TNSS and VAS scores were significantly higher during the exposure period than those during the off-exposure period (Figure 1A,B). Large effect sizes were observed for both comparisons (*r_rb_* = 0.78 and *r_rb_* = 0.52, respectively). Both timothy grass allergen-specific and total IgE concentrations were significantly lower during the exposure period than during the off-exposure period (Figure 1C,D). A large effect size was observed for timothy grass allergen-specific IgE (*r_rb_* = 0.53), and a moderate effect size (*r_rb_* = 0.39) for total IgE when comparing the exposure and off-exposure periods in this group. Our results also showed significantly higher median sIgE and total IgE levels in AR participants than in LAR participants during the exposure period (Figure 2A,B). For the comparisons between the LAR and AR groups during the exposure period, a large effect size (*r_rb_* = 0.70) was observed in the case of timothy grass pollen-specific IgE and a moderate effect size (*r_rb_* = 0.44) in the case of total IgE. The TNSS and VAS scores, sIgE levels for timothy grass, and total IgE levels in nasal lavage fluid for the LAR and AR groups are presented in Table 2.

Of the 15 NPTs performed, seven were positive, further supporting the diagnosis of LAR. Subjects with positive NPT were designated as group A, and those with negative NPT as group B. In group A, the median TNSS score was significantly higher post-provocation (median = 4 [IQR 2–4]) compared to pre-NPT (1; [IQR 1–2]) (*p* = 0.03), with a large effect size (*r_rb_* = 0.94). The median VAS score in group A was also higher post-NPT (45; [IQR 31–63]) compared to pre-NPT (28; [IQR 1–25]) (*p* = 0.03), also with a large effect size (*r_rb_* = 0.86). For group B subjects, the median TNSS score was similar pre- and post-NPT, whereas the median VAS score was lower post-NPT, with no significant difference observed (Table 3).

Regarding sIgE levels, the median level was lower (but not significantly) post-NPT in group A (0.2023; [IQR: 0.1287–0.2357]) than pre-NPT (0.2312; [IQR:0.1505–0.2357]). Similarly, a non-significantly lower median level of sIgE to g6 was noted in group B post-NPT (0.2903; [IQR:0.2135–0.4538]) compared to the pre-NPT level (0.2977 [IQR: 0.2609–0.4436]). Regarding total IgE levels in nasal secretions, a significantly higher level was observed pre-NPT than post-NPT in subjects with negative NPT results (group B), with a large effect size (*r_rb_* = 0.92) (Table 4).

**Figure 3:**
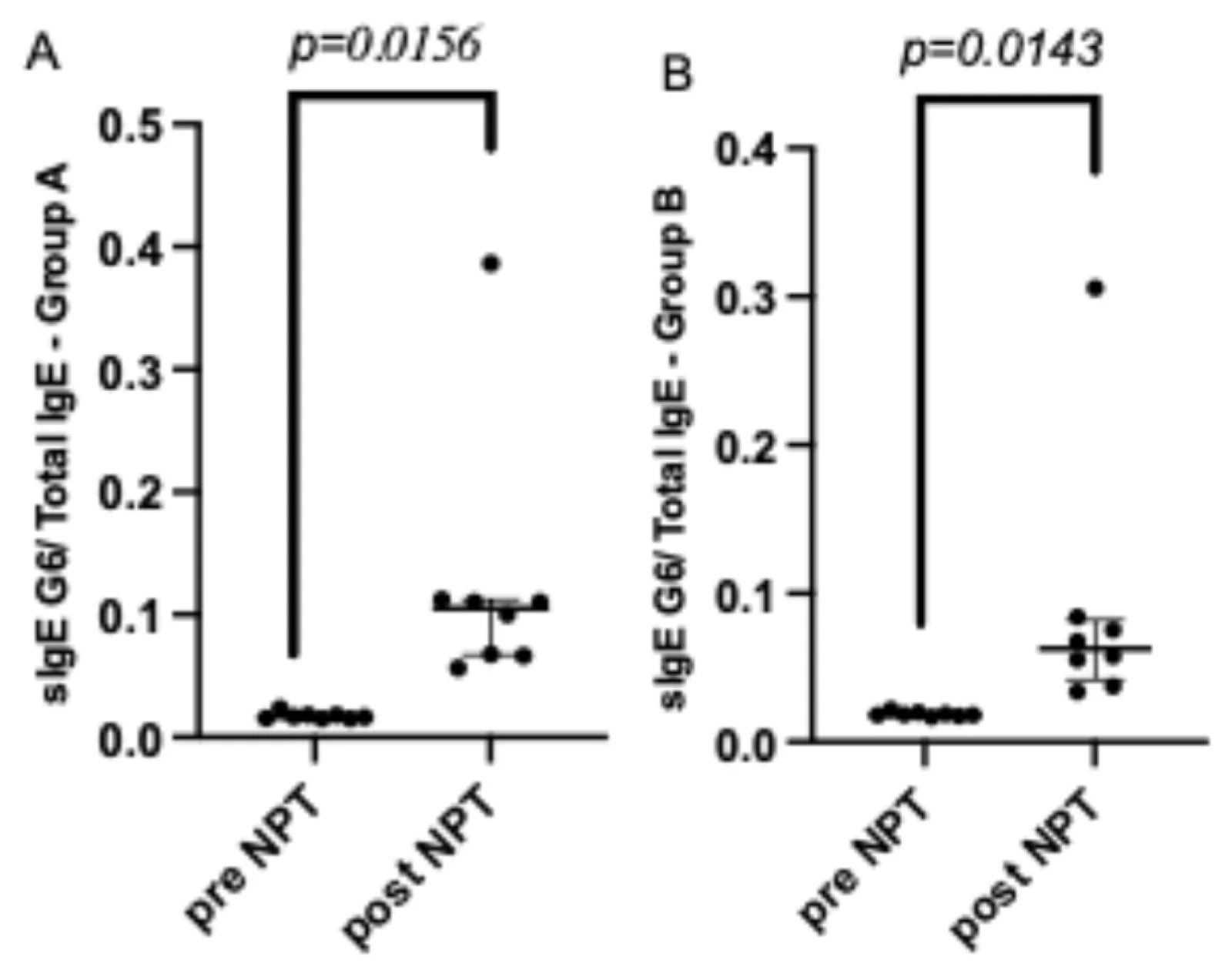
Comparison of relative sIgE levels to timothy grass pollen calculated as sIgE/total IgE before and after provocation in Group A (**A**) and Group B (**B**). NPT: nasal provocation test; sIgE: specific immunoglobulin E; G6: Timothy Grass. The results are presented as median + interquartile range (IQR).

Regarding relative sIgE concentration calculated as sIgE/total IgE, our study showed a significantly higher median relative sIgE level after provocation (0.1003; IQR 0.06642–0.1124; 95%CI 0.06–0.39) as compared to pre-NPT value (0.01627; IQR 0.01536–0.01826; 95%CI 0.015–0.024) (*p* = 0.0156), with a large effect size (*r_rb_* = 0.93). (Figure 3A). Similarly, in Group B, a significantly higher median relative sIgE concentration was observed post-NPT (0.06290; IQR 0.04193–0.08201; 95%CI 0.03–0.31) compared to that before NPT (0.01825; IQR 0.01772–0.01962; 95%CI 0.017–0.022) (*p* = 0.0143), with a large effect size (*r_rb_* = 0.92) (Figure 3B).

**Figure 4:**
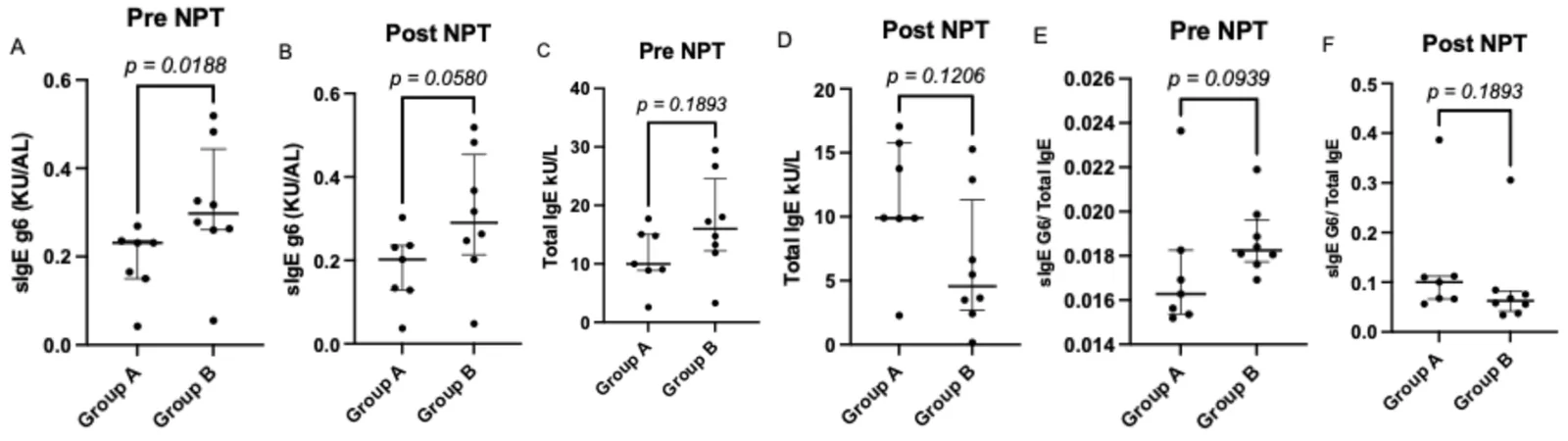
Comparison of sIgE g6 (**A**,**B**), total IgE (**C**,**D**), and relative sIgE levels (**E**,**F**) for timothy grass pollen between Groups A and B before and after nasal provocation. NPT, nasal provocation test; sIgE, specific immunoglobulin E; G6, Timothy Grass. Each dot represents one sample. Medians with interquartile ranges (IQR) are indicated.

Our results showed a higher total IgE level in the positive NPT group than in the negative NPT group post-provocation (Figure 4D); however, pre-NPT, the total IgE level was lower in the positive NPT group (Figure 4C). Additionally, a higher median relative sIgE-g6 change was noted in Group A only post-provocation compared to Group B (Figure 4E,F). Surprisingly, a higher level of sIgE to timothy grass was observed in the negative NPT group, both before and after provocation, compared to the positive NPT group (Figure 4A,B). Statistical significance was reached solely in the case of pre-NPT comparisons (Figure 4A), with an *r_rb_* value of 0.59 indicating a large effect size and potentially meaningful difference.

### 3.3. Grass Pollen Counts During the Study Period

**Figure 5:**
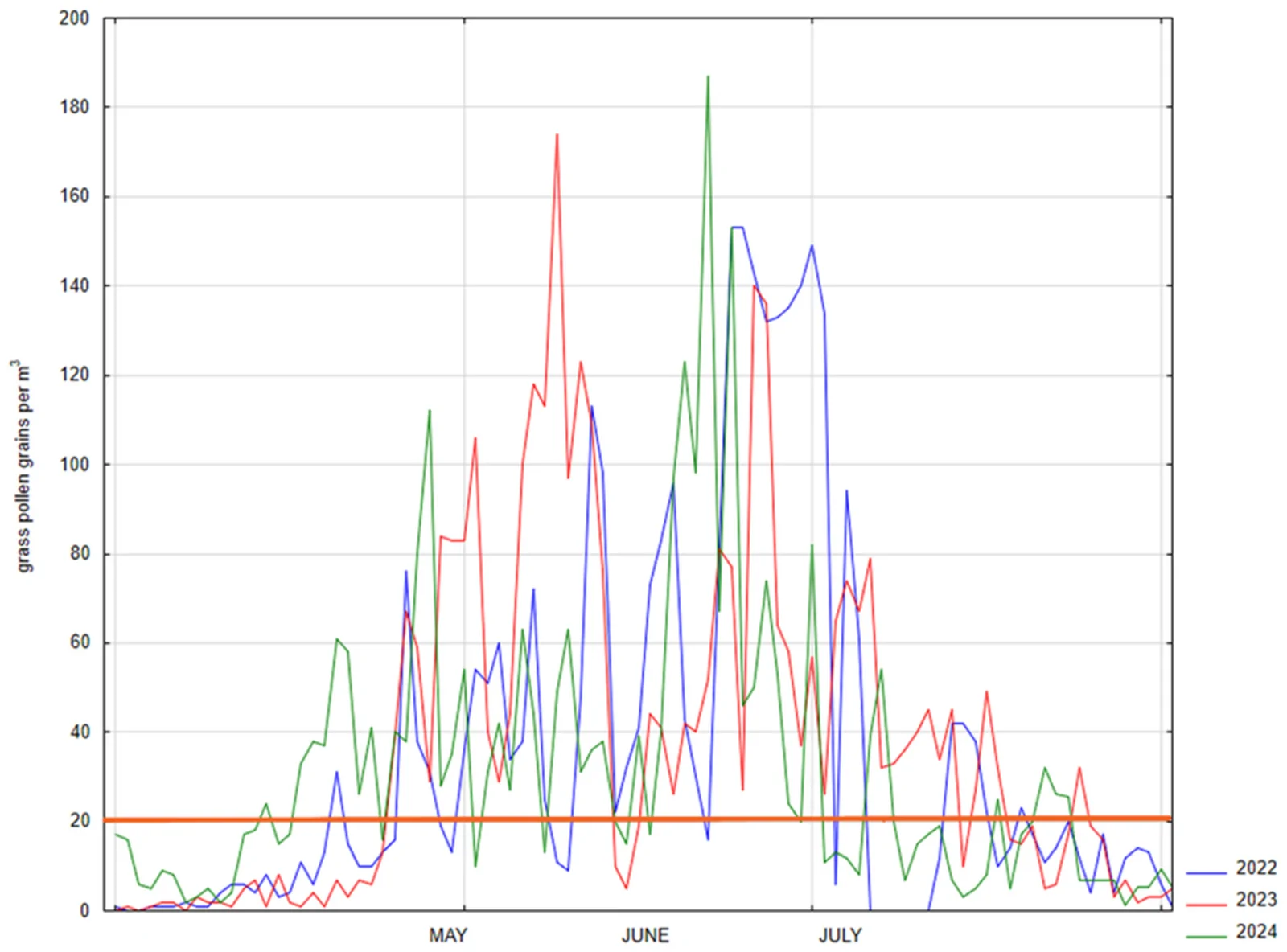
Grass pollen counts (grains per cubic meter) in the study area during the pollination seasons of 2022–2024. The orange line indicates the threshold of 20 pollen grains per m^3^, which is considered clinically important as a trigger for allergy symptoms in sensitive individuals.

Data on grass pollen counts in the study area during the pollination season in 2022, 2023, and 2024 are presented in Figure 5.

Throughout the season, the quantity of grass pollen grains in the atmosphere exceeded the threshold of 20 grains/m^3^, which was previously assessed as causing allergic symptoms in the sensitized population of Poland [17]. During the seasons when participants were recruited, a high degree of exposure to grass pollen was observed, following a similar pattern in subsequent years (Figure 5). Grass pollen counts did not differ significantly between seasons [15.5 (6.0–46.75) grains/m^3^ in 2022; 28 (5.0–58.75) grains/m^3^ in 2023; 20 (8.25–40.75) in 2024; median (interquartile range); *p* = 0.53, Kruskal–Wallis test].

## 4. Discussion

Diagnosing and managing LAR subjects remain significant challenges for physicians. A considerable number of patients with LAR are still either underdiagnosed or misdiagnosed as having non-allergic rhinitis. Negative SPT and serum-sIgE results make these methods ineffective for diagnosing LAR. To date, the gold-standard method for diagnosing LAR subjects is nasal provocation [23]; however, this method is time-consuming and challenging because it must be performed by trained personnel.

Our study showed that TNSS and VAS scores were higher during the grass pollen allergen exposure period than during the off-exposure period, consistent with the natural exposure effect of allergens on suspected LAR patients. Despite a significant increase in subjective assessment scores in participants with suspected LAR and AR during the timothy grass allergen exposure period, a slightly lower median sIgE level to g6 was observed in both groups during the exposure period compared to the off-exposure period. In contrast, a higher median total IgE level was observed only in the AR group during the exposure period.

In our study, local allergic rhinitis to grass pollen was confirmed in 46.6% of participants. Both TNSS and VAS scores were significantly higher after positive nasal provocation. Clinically, low TNSS and VAS scores were observed in LAR participants following negative nasal provocation. The subjective questionnaire is useful for pre- and post-provocation assessments; however, its use alone may affect the final evaluation of post-NPT manifestations in patients with local allergic rhinitis. In a study by Rondon et al., NPT with grass pollen was performed in 60 subjects (30 with LAR and 30 healthy controls), and positive nasal provocation was noted among all LAR subjects. An increase in sIgE to grass pollen was observed in 30% of LAR subjects, with a significant elevation at 6 and 24 h post-provocation [3]. In our study, a greater relative change in sIgE was observed post-provocation among subjects with a confirmed LAR diagnosis and among those with a negative NPT. In a similar study conducted in Spain by Blanca Lopez et al., 61 patients with seasonal rhinitis (negative SPT and serum IgE) were included, and nasal provocation with Phleum extract was performed. LAR was confirmed in 61% of the subjects, and among these, an increase in sIgE to Phleum was observed 15 min after provocation, with the greatest increase at 1 h post NPT [24].

In another study conducted in Poland in 2016, 121 subjects with non-allergic rhinitis (12–18 years) were recruited, and nasal provocation with grass, mugwort, and birch pollen was performed. LAR to grass pollen was confirmed in approximately 16.6% of the subjects, and it was observed that the sIgE level in nasal secretion increased among LAR patients’ post-provocation [25]. In a similar study, however, assessing suspected house dust mite-sensitized LAR patients, 181 chronic rhinitis subjects were included. Nasal provocation was then performed and the concentration of local sIgE of *Dermatophagoides pteronyssinus* and *Dermatophagoides farinae* as well as a panel of cytokines including Interleukin-4, Interleukin-5, C-C motif chemokine ligand 5 (CCL5), and C-C motif chemokine ligand 11 (CCL11) were assessed in nasal secretions [26]. The results of the study showed that subjects with higher sIgE levels had significantly higher concentrations of type 2 cytokines; thus, a positive correlation could be established between local sIgE production and the increase in type 2 cytokines post-provocation among suspected LAR subjects [26].

The percentage of LAR subjects varied between the studied populations. As we have seen in previously mentioned studies, the concentration of sIgE in nasal secretions varies post-provocation across populations and methodologies [27]. In our study, for example, LAR to grass pollen was confirmed in 46.6% of the suspected patients, but sIgE and total IgE concentrations were relatively low post-provocation. The low sIgE and total IgE levels may be due to several factors. First, we collected nasal secretions through the nasal lavage method 15 min post-provocation, which could have prevented a potential increase in the measured levels if nasal secretions were taken after 1 and 6 h, as in the study of Rondon et al. [3]. Secondly, a possible decrease in sensitivity because of dilution in the collected nasal lavage fluids. Therefore, in our study, we corrected for dilution by calculating the dilution factor from comparisons of urea concentrations in the serum and nasal lavage fluid [17,28,29]. The use of urea concentration in serum and in lavage material in the formula calculating dilution factor has long been considered as one of the most useful methods for standardization of protein concentrations in nasal secretions, and its use has been established and justified already in the late 1980s [30,31]. For this purpose, urea has been chosen a standardization parameter, due to the stability of its concentration in the nasal secretions and close correlation of concentrations in NLF with those in serum. However, studies on nasal secretions in LAR also differ with regard to the dilution method chosen (if any). Therefore, one of the main challenges in this context is to establish a defined correction protocol for the dilution effect on sIgE assessment in nasal secretions.

### Limitations

Although our study showed promising results in assessing sIgE in nasal secretions of LAR subjects, several limitations were noted. First, a relatively small number of subjects were included, which affected generalizability and limited the study’s statistical power. Nevertheless, the reported large effect sizes further support the assumption that shifts and differences in NLF IgE levels are of practical significance. Effect sizes for non-parametric tests may tend to be larger when smaller samples are analyzed [32]. In this context, especially the unexpectedly lower IgE levels noted post-provocation, require further studies to be confirmed.

Second, objective assessments, such as rhinomanometry, were not used in the study, and confirmation of LAR diagnosis was based on the subjective assessment of TNSS and VAS. One of the main limitations of our study was the relatively low level of sIgE to timothy grass after nasal provocation. This may be due to several factors; first, the short time interval between provocation and nasal secretion acquisition (limited to 15 min) could have affected the measured sIgE concentration. The dilution effect is another major factor affecting the measured sIgE level. Thus, in our study, a lower absolute sIgE level was noted post-provocation in the group of subjects in whom LAR diagnosis was confirmed. The sIgE/total IgE ratio helps interpret sensitization in allergy diagnosis, especially in predicting outcomes in patients undergoing allergen immunotherapy [33]. In our study, we used the sIgE/total IgE ratio, which was also significantly higher after provocation in both the positive and negative NPT groups. The increase in this ratio correlates with the positive subjective assessment using TNSS and VAS. Although this ratio has not yet been included in the diagnostic workup of LAR, it can be used as an add-on tool in the assessment of LAR subjects.

Additionally, non-IgE-mediated rhinitis symptoms can be elicited by natural exposure to allergens and after nasal provocation, which further blurs the clinical picture and challenge test outcomes. This fact deserves attention in light of the new classification and nomenclature of hypersensitivity reactions, recently proposed by the European Academy of Allergy and Clinical Immunology (EAACI) [34]. In a study published by Olivier et al. in 2015, it was demonstrated that the combination of NPT with objective assessment tools, such as spirometry or peak expiratory flow (PEF) measurements, helps in the assessment of both nasal and bronchial allergen-induced hyperresponsiveness [35], which supports the use of NPT to confirm clinically relevant allergy before the prescription of allergen immunotherapy. In a recently published study by the same author, it was found that tests assessing and characterizing cellular immunoreactivity to pollen and citrus allergens in patients with non-IgE-mediated rhinoconjunctivitis, such as the Leukocyte Adherence Inhibition Test (LAIT) and Tube Titration of Precipitins (TTP), may serve as an adjunctive tool for endotyping and assessing allergen cross-reactivity, with LAIT slightly outperforming TTP [36].

## 5. Conclusions

The assessment of local sIgE levels in nasal secretions can help in the diagnostic workup of patients with local allergic rhinitis. Our results suggest that further investigation in larger cohorts of well-characterized patients with confirmed local allergic rhinitis is warranted. The small sample size in our study precluded an effective assessment of this method’s clinical relevance; larger studies are needed in this context. A future goal is to develop a well-defined and standardized protocol for assessing sIgE concentrations in nasal secretions from patients with local allergic rhinitis.

## Figures and Tables

**Table 1:** Descriptive data of the subjects.

Subjects (N = 29)	Gender	Age + [Range] in Years
LAR (n = 18)	F (n = 11)	47 (19–69)
	M (n = 7)	32 (23–57)
AR (n = 11)	F (n = 5)	20 (18–68)
	M (n = 6)	27 (19–44)

LAR, Local allergic rhinitis. AR, Allergic Rhinitis. Results are presented as median + interquartile range (IQR).

**Table 2:** Comparison of TNSS, VAS, and sIgE to g6 and Total IgE between the exposure period and off-exposure period to grass pollen allergen in LAR and AR subjects.

		LAR	AR	*p* Value
TNSS [0–12]	EP	6; [IQR: 4–7; 95%CI 3–8]	4.5 [IQR: 4–7.25; 95%CI 4–8]	*p* = 0.9256
	OEP	1 [IQR: 0–2; 95%CI 0–2]	n.d	N. A
VAS [0–100]	EP	50 [IQR: 32–70; 95%CI 26–76]	50 [IQR; 25–65; 95%CI 30–82]	*p* = 0.9043
	OEP	21 [IQR: 1–34; 95%CI 1–34]	n.d	N. A
sIgE g6 (kUA/L)	EP	0.1260 [IQR: 0.1168–0.1663; 95%CI 0.11–0.19]	0.2897 [IQR: 0.2034–0.3752; 95%CI 0.16–0.39]	*p* = 0.0008
	OEP	0.2601 [IQR: 0.1655–0.3173; 95%CI 0.17–0.32]	n.d	N. A
Total IgE (kU/L)	EP	8.044 [IQR: 6.946–10.97; 95%CI 6.89–12.82]	12.73 [IQR: 9.266–16.58; 95%CI 8.22–16.67]	*p* = 0.0491
	OEP	14.74 [IQR: 9.063–17.73; 95%CI 9.06–17.73]	n.d	N. A

LAR, Local allergic rhinitis. AR, Allergic Rhinitis. TNSS, Total nasal symptom score; VAS, Visual analog scale; sIgE, specific immunoglobulin E; g6, Timothy Grass. EP, Exposure period. OEP, outside of exposure period; 95%CI, 95% confidence interval of the median; n.d, not done; N. A, not applicable. Results are presented as medians with interquartile ranges (IQR) and 95%CI in square brackets.

**Table 3:** Comparison of TNSS and VAS scores before and after provocation and in suspected LAR subjects.

	TNSS			VAS		
	Pre-NPT	Post-NPT	*p* Value	Pre-NPT	Post-NPT	*p* Value
Group A n = 7	1 [IQR: 1–2; 95%CI 0–2]	4 [IQR: 2–4; 95%CI 2–7]	*p* = 0.03	28 [IQR: 1–52; 95%CI 0–65]	45 [IQR: 31–63; 95%CI 23–75]	*p* = 0.03
Group B n = 8	1 [IQR: 0–1.75; 95%CI 0–6]	1 [IQR: 0–1; 95%CI 0–2]	*p* = 0.5	16 [IQR: 1.5–26.5; 95%CI 0–74]	11 [IQR: 0.75–22; 95%CI 0–77]	*p* = 0.3438

LAR, local allergic rhinitis; TNSS, total nasal symptom score; VAS, visual analog scale; NPT, nasal provocation test; 95%CI, 95% confidence interval of the median; Results are presented as medians with interquartile ranges (IQR) and 95%CI in square brackets.

**Table 4:** Comparison of sIgE to Timothy grass pollen allergen extract (g6) and total IgE pre- and post-nasal allergen provocation in suspected LAR subjects.

	sIgE g6			Total IgE		
	Pre-NPT	Post-NPT	*p* Value	Pre-NPT	Post-NPT	*p* Value
Group A n = 7	0.2312 [IQR: 0.1505–0.2357; 95%CI 0.04–0.27]	0.2023 [IQR: 0.1287–0.2357; 95%CI 0.04–0.30]	*p* = 0.7360	9.969 [IQR: 8.894–15.06; 95%CI 2.59–17.73]	9.910 [IQR: 9.850–15.75; 95%CI 2.27–17.06]	*p* = 0.9999
Group B n = 8	0.2977 [IQR: 0.2609–0.4436; 95%CI 0.06–0.52]	0.2903 [IQR: 0.2135–0.4538; 95%CI 0.05–0.52]	*p* = 0.7801	16 [IQR: 12.22–24.55; 95%CI 3.27–29.44]	4.560 [IQR: 2.675–11.33; 95%CI 0.16–15.27]	*p* = 0.0207

LAR, local allergic rhinitis; sIgE, specific immunoglobulin E; NPT, nasal provocation test; g6, Timothy grass pollen allergen extract. Results are presented as median (IQR).

## Data Availability

The original contributions presented in this study are included in this article. Further inquiries should be directed to the corresponding author.
